# The Interplay of Lung Cancer, COVID-19, and Vaccines

**DOI:** 10.3390/ijms232315067

**Published:** 2022-12-01

**Authors:** Dragan Trivanović, Željka Peršurić, Andrea Agaj, Marko Jakopović, Miroslav Samaržija, Lela Bitar, Krešimir Pavelić

**Affiliations:** 1 Department of Oncology and Hematology, General Hospital Pula, Santorijeva 24a, 52100 Pula, Croatia; 2 Faculty of Medicine, Juraj Dobrila University of Pula, Zagrebačka 30, 52100 Pula, Croatia; 3 Faculty of Chemical Engineering and Technology, University of Zagreb, Trg Marka Marulića 19, 10000 Zagreb, Croatia; 4 Department for Respiratory Diseases Jordanovac, KBC Zagreb-Jordanovac Clinic for Lung Diseases, 10000 Zagreb, Croatia

**Keywords:** COVID-19, vaccine, solid cancers, lung cancer, immune checkpoint inhibitors

## Abstract

Patients with cancer are more susceptible to a higher risk of coronavirus infection and its severe complications than the general population. In addition, these patients were not included in the pivotal clinical trials for COVID-19 vaccines. Therefore, considerable uncertainty remains regarding the management of cancer patients during the COVID-19 pandemic and the safety of COVID-19 vaccinations in cancer patients. In this review, we summarize the current knowledge generated from the beginning of the COVID-19 pandemic on the vulnerability of cancer patients to the coronavirus disease, as well as the effectiveness of COVID-19 vaccines in this population. We also discuss the available data on the effects of anticancer treatment with immune checkpoint inhibitors on the immune responses to SARS-CoV-2 in cancer patients. Special attention in this review will be given to patients with lung cancer, as such patients are at an increased risk for severe effects from COVID-19.

## 1. Introduction

The Coronavirus disease 2019 (COVID-19) pandemic has negatively affected the treatment of malignant diseases and the development of new prodrugs worldwide. The extent of these negative effects is still not possible to estimate. A survey of 116 U.S. lung cancer screening programs found that most of the programs (85%) were seriously delayed or even stopped for at least 5 weeks due to the COVID-19 pandemic [1]. A study on U.S. patients found that the number of patients diagnosed with cancer significantly decreased during the early pandemic period (1 March 2020–18 April 2020) compared to the pre-pandemic period (6 January 2019–29 February 2020) [2]. As a result of lung-cancer-specific screening delays and scheduling problems, researchers are expecting a 5% increase in lung cancer mortality in the United Kingdom over the next 5 years [3]. Another study found an increase in the percentage of patients with potentially malignant lung nodules (29% versus 8%) [4]. However, despite the pandemic, there are also examples of new screening programs. In Croatia, the first nationwide lung cancer screening program in Europe was initiated during the pandemic [5]. After 18 months of the program, more than nine thousand people at risk were screened, with response rates of more than 90% after invitation by general practitioners.

The COVID-19 pandemic also affected basic cancer science and even more clinical trials included poor patient enrolment during the pandemic [6]. From March to May 2020, the number of patients enrolled in clinical trials for the Alliance for Clinical Trials in Oncology dropped by more than half. Improvements were not observed until the fall of 2020 [1].

The scientific community is now seeking answers to many questions that arise from the complex interplay between cancer and COVID-19.

Furthermore, the influence of cancer therapy on infection risk and the influence of vaccinations on cancer therapy are not yet well understood. The vast majority of articles deal with the efficiency and safety of the use of vaccines in lung cancer patients. At the same time, there is a significant gap in the literature on the complex relationship between the influence of COVID-19 pneumonia and the vaccine itself in the growth of lung cancer and treatment complications. Therefore, a clear challenge has arisen: the need to review which of these relationships can be analyzed from another perspective. In this review, we will outline recent knowledge on the interplay between COVID-19 infection, cancer treatment outcomes, and vaccine efficacy. Special focus will be placed on lung cancer and immunotherapy, as higher risks specific to certain cancer diagnoses or regimens could be observed.

## 2. COVID-19 and Lung Cancer

Studies show that patients with a variety of comorbidities (e.g., hypertension, diabetes, and obesity) are at a higher risk of hospitalization due to COVID-19 compared to those without such conditions [7]. Cancer patients are also considered a particularly vulnerable population during this global pandemic due to their systemic immunosuppressive state caused by malignancy and anticancer treatments. The earliest studies indicated that patients who have other underlying chronic conditions were at a higher risk than cancer patients of becoming critically ill if infected with severe acute respiratory syndrome Coronavirus 2 (SARS-CoV-2) [8]. However, the early data also showed that patients with cancer might have a higher risk of COVID-19 than individuals without cancer, as well as a higher risk of severe events (intensive care unit admission, invasive ventilation, or death) compared to patients without cancer [9,10,11]. Unfortunately, these early reports are restricted by sample size and geographical region, as well as a lack of generalizability of the study results to the overall population of patients with cancer. To compensate for the limitations of these early studies on the effects of cancer among patients with COVID-19, Yang et al. performed a meta-analysis of 19 retrospective studies that included 63,019 patients with COVID-19 across nine countries [12]. The pooled incidence of cancer in COVID-19 patients was 6%, which was much higher than the global cancer incidence of approximately 0.2%. It also remains unclear whether patients with cancer might be immunocompromised by the effects of antineoplastic therapy, supportive medications such as steroids, and the immunosuppressive properties of cancer itself [13].

Diagnosing COVID-19 in patients with cancer is another challenge due to multiple factors [14]. One of these factors is that cancer patients might have atypical radiographic features [15] or radiographic findings similar to those of a SARS-CoV-2 infection, which can be misleading [16]. Moreover, it is important to note that due to similar symptoms between the infection and the underlying disease, the diagnosis of COVID-19 may be delayed, particularly in lung cancer patients and patients with pulmonary metastasis. Additionally, some clinical and biological characteristics can mask COVID-19 presentation in cancer patients [17]. On the other hand, COVID-19 may delay the initial diagnosis and treatment of lung cancer. The immune activation required for antitumor immune responses sometimes triggers autoimmunity and immunorelated adverse events (irAE) [18,19,20]. Pneumonitis is a rare but serious irAE complication in patients receiving immune checkpoint inhibitors (ICIs). The radiological discovery of SARS-CoV-2 infection in lung cancer may overlap and be confused with lung cancer progression or immune-related pneumonitis as a complication of anticancer therapies [21]. Thus, interpreting lung cancer’s initial diagnosis or clinical status may, in some situations, be challenging and harbor the possibility of misdiagnosis [22,23,24,25,26,27].

Patients with lung cancer are especially vulnerable to the Coronavirus disease 2019, as a higher rate of becoming infected compared to other patients with cancer was previously reported. The reasons for this increased vulnerability still need to be elucidated. An Italian study that estimated the rate of SARS-CoV-2 infection among approximately 60,000 patients receiving antitumor treatment found that patients with lung cancer had the highest incidence of contracting COVID-19 (n = 91, 22.4 %) [28]. Case-control analysis of risks and outcomes among US patients with cancer and COVID-19 infection showed that patients recently diagnosed with lung cancer were at a significantly higher risk of infection with SARS-CoV-2 (OR 7.66 (95% CI 7.07–8.29) [29].

Patients with lung cancer also have higher mortality and hospitalization rates with more severe outcomes compared to other patients with cancer (Table 1). Possible reasons include a notable overlap in respiratory symptoms between lung cancer and COVID-19, with theoretically synergistic morbidities. Additionally, many patients with lung cancer undergo immunosuppressive cancer therapies [6].

As a global consortium, the Thoracic Cancers International COVID-19 Collaboration (TERAVOLT) registry was formed to determine the connection between demographics, clinical characteristics, and outcomes in patients with lung cancer and COVID-19. Univariable logistic regression analysis of 200 patients with COVID-19 and lung cancer revealed associations linking increased mortality with an age greater than 65 years, smoking, chemotherapy treatment, and the presence of comorbidities. However, in multivariable analyses, only smoking history was statistically associated with an increased risk of death [30]. The results presented in all these studies confirmed that patients with lung cancer are among the most vulnerable populations to COVID-19.

**Table 1:** Mortality and morbidity in patients with SARS-CoV-2 infection and lung cancer.

Population	Number of Patients with Lung Cancer	Hospitalization among Patients with Lung Cancer	Mortality among Patients with Lung Cancer	Reference
Adult patients with solid or hematological malignancies	1135	-	CFR 32.4%	[31]
Patients with lung cancer from a single center in New York City	102	63 (62%)	25 (25%)	[32]
Patients with thoracic cancers from eight countries	200	152 (76%)	66 (33%)	[30]
Patients from 14 hospitals in China	22	-	4 (18%)	[9]
Patients from the Veneto region (Italy)	21	13 (62%)	5 (24%)	[33]
Adult patients with cancer enrolled in the UK Coronavirus Cancer Monitoring Project (UKCCMP)	111	-	43 (39%)	[34]
Patients included in the Dutch Oncology COVID-19 Consortium registry	47	-	22 (47)	[35]

CFR—case-fatality rate, defined as the rate of death in this population.

**Figure 1:**
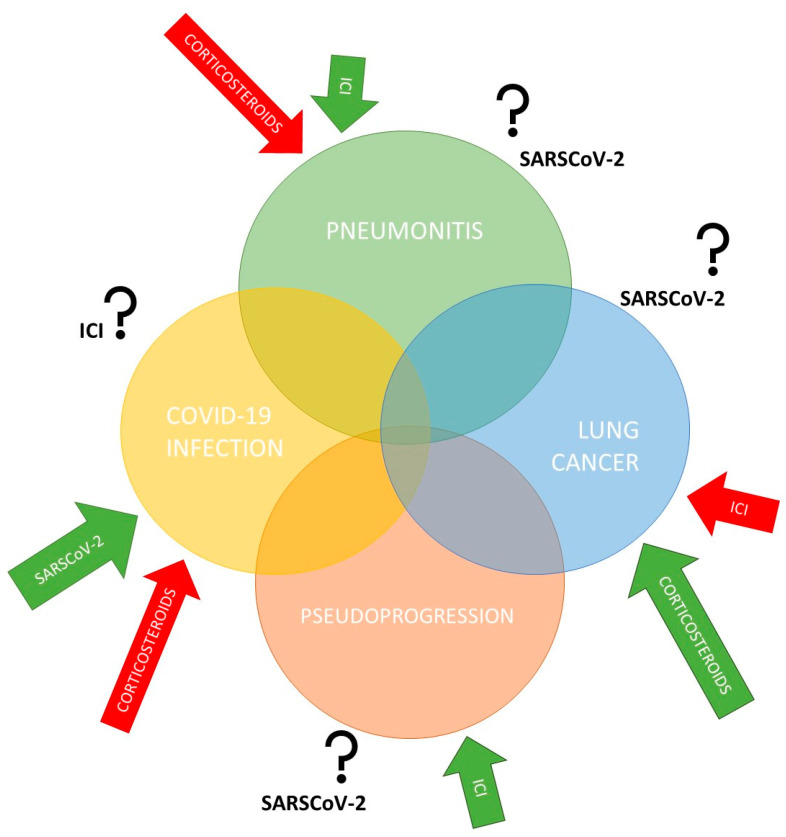
The interplay between COVID-19 infection, lung cancer, pneumonitis, and pseudoprogression. Lung cancer is characterized by an increased risk of pulmonary complications, as is COVID-19 infection, due to pathophysiological, clinical, and treatment-related risk factors. In addition, there remains concern regarding overlapping pneumonitis from immune checkpoint inhibitors (ICIs) and COVID-19-induced pneumonia. Therefore, the influence of specific treatments and SARS-CoV-2 virus on each segment still needs to be clarified.

The lungs are the most strongly affected organs in SARS-CoV-2 infection. Additionally, within the lungs, as in other human organ tissues, angiotensin-converting enzyme 2 (ACE2) was proven to be the main host cell receptor for the binding of SARS-CoV-2 [36]. ACE2 expression is also elevated in tumor and tumor-adjacent normal tissues in patients with lung cancer [37,38], which might partially explain why patients with lung cancer are potentially at a higher risk of severe COVID-19. ACE2 expression patterns and levels are closely associated with a susceptibility to and symptoms of COVID-19 [39]. Moreover, tumor cells are more susceptible to viral replication due to defects in innate antiviral immunity associated with transformation [40]. However, there is still a need to clarify the factors affecting SARS-CoV-2 replication in lung cancer cells, as well as the possible impact of COVID-19-induced inflammation on lung cancer development and its pathophysiology (Figure 1). The positive impact of immunotherapy on enhancing the antitumor response was clearly demonstrated, as was the negative impact of corticosteroids in the same process. For this reason, the concomitant application of corticosteroids with ICIs is avoided [18]. The influence of corticosteroids in the treatment of the COVID-19 infection and serious side effects such as pneumonitis were also shown to have significant effects and is an integral part of the COVID-19 treatment protocol [21,24,25]. The simultaneous mutual effects between all these factors, however, have not been sufficiently investigated or have provided conflicting results, especially with anti-COVID-19 vaccination. Cancer cells require high energy to survive and can recapture mitochondria from the microenvironment, including from immune cells. The abundance of mitochondria potentially represents a good environment for high replication of SARS-CoV-2 [41,42].

## 3. COVID-19 and Anticancer Treatment with Immune Checkpoint Inhibitors

Over the last decade, immune-oncology (IO), specifically ICIs, has become one of the most promising areas of cancer research, as well as one of the fastest-growing areas of drug development, with the approval of more than 20 agents globally [43]. Data strongly support the concept that the immune system can identify, locate, and control tumor cells in a process called cancer immunosurveillance. In addition, the immune system can cooperate with the tumor microenvironment and promote tumor progression through chronic inflammation, the immunoselection of poorly immunogenic variants, and immunosuppression [44,45].

Since ICIs can change immune competence, it is important to clarify whether the immune response to SARS-CoV-2 is influenced by patients receiving ICIs or other immunomodulating treatments, including steroid therapy [46]. To date, most studies have not found an association between cancer therapy and increased mortality among patients with cancer and COVID-19 [47]. An analysis by the COVID-19 and Cancer Consortium (CCC19) of 928 patients with cancer and COVID-19 alongside 91 patients with thoracic malignancies and COVID-19 confirmed that there is no connection between 30-day all-cause mortality and recent surgery, recent noncytotoxic therapy, or recent cytotoxic therapy (mostly chemotherapy) [48]. The later analysis of almost 5000 patients with cancer and COVID-19 reported to the CCC19 confirmed that immunotherapy alone was not associated with higher COVID-19 severity [47]. Other studies did not find any statistically or clinically significant relationship between steroid treatment, ICIs treatment, and COVID-19 outcomes. These data are mostly from unvaccinated patients, and it should be noted that these studies were not explicitly designed to assess this effect [30,32].

There is a concern about the possible negative interference of ICIs in the severity of COVID-19. It is known one of the most important mechanisms underlying the progression of COVID-19 is cytokine storm. It is very important to recognize cytokine storm because it has negative prognostic and therapeutic implications and can lead to acute respiratory distress syndrome, multiple organ failure, and deterioration of cancer treatment outcomes [49]. Cytokine storm and cytokine release syndrome (CRS) are life-threatening systemic inflammatory syndromes involving elevated levels of circulating cytokines and immune-cell hyperactivation that can be triggered or worsened by various therapies, including ICIs, chemotherapy, other pathogens, autoimmune conditions, and comorbidity. Cytometric analyses of COVID-19 patients showed reduced counts and a hyperactivated status of peripheral CD4 and CD8 T cells. Furthermore, studies found an increased concentration of proinflammatory T cells. Additionally, CD8 T cells were found to harbor high concentrations of cytotoxic granules, suggesting that the hyperactivation of T cells worsens autoimmune injury [50]. Considering this immunological interplay, the hypothesis of a correlation between ICIs mechanisms, tumor immunology, and irAE alongside COVID-19 pathogenesis should be considered. Notably, ICIs-induced CRS is rare, and a cytokine storm is a late event in the COVID-19 pathogenesis. Moreover, patients still receive ICIs during the early stages of COVID-19 infection [46].

## 4. COVID-19 Vaccines in Patients with Cancer

Studies on more than 1000 cancer patients have investigated the COVID-19 vaccine’s safety and effectiveness, including the early safety profile of the BNT162b2 vaccine in 134 patients with cancer (including lung cancer) under immune checkpoint blockage [51,52,53,54,55,56,57]. The most common side effects were mild and similar to those of other common vaccines [58]. Initial data suggest that SARS-CoV-2 vaccines are effective in patients with cancer (Table 2). However, most studies only assessed seroconversion in patients with cancer, and only some studies performed neutralization assays to measure neutralizing antibody responses against variants of concern [59,60].

Moreover, patients with solid and hematologic cancer were not included in pivotal clinical trials conducted to demonstrate the efficacy and safety of COVID-19 vaccines. Patients with cancer appear to be more likely to develop a less efficient immune response following vaccination against COVID-19. Prior to the predominance of the Omicron variant, this ability was assessed to be lower than that in immunocompetent individuals [56,68,70,71,72,73,74,75]. Embi et al., in a large study including only patients with cancer who received mRNA vaccines, showed that the efficacy was about 60%, with no further reduction observed; although, there was a high prevalence of the Delta variant in the sample [68]. However, based on additional studies, we can conclude that the majority (90–100%) of patients with solid tumors seroconvert after two vaccine doses, and the growing data suggest that antibody titers are comparable to those in individuals without cancer or simply less reduced [53,54,56,59,60,73,74]. Data from studies also suggest that COVID-19 vaccines could potentially be less effective in patients with lung cancer than in those with other solid tumors and healthy controls. Massarweh et al., in an Israeli study, showed that patients with lung cancer had lower antibody levels compared to healthy individuals and the overall solid cancer cohort [53].

Risk factors that can impair seroconversion in patients with solid tumors include older age, male sex, vaccine type, and chronic corticosteroid use. The influence of chemotherapy and immunotherapy on the efficacy of COVID-19 vaccines is of special interest. Recent chemotherapy has been repeatedly identified as a strong risk factor for lower seroconversion and neutralizing responses [59,76,77]. Oosting et al. recently reported the impact of immunotherapy, chemotherapy, and chemoimmunotherapy on the immunogenicity and safety of the COVID-19 vaccination in patients treated for solid tumors. The study confirmed that the mRNA-1273 vaccine is safe and effective in patients with cancer, including lung cancer. Nevertheless, a significant minority of the patients did not develop an adequate antibody response (6.9% in the immunotherapy cohort, 16.2% in the chemotherapy cohort, and 11.2% in the chemoimmunotherapy cohort) [66]. Similar protection rates against severe COVID-19 were confirmed in real life by population-based Israeli and Scottish studies for the Pfizer BNT162b2 and AstraZeneca ChAdOx1nCov-19 vaccines [4,78]. A lack of immunization was associated with age, corticosteroid treatment, and cytotoxic chemotherapy.

Furthermore, the safety and immunogenicity of one versus two doses of the COVID-19 vaccine for patients were analyzed. Monin et al. found poor vaccine efficacy among patients with cancer after the first dose of BNT162b2 (38% seropositivity in patients with solid tumors versus 94% for the healthy controls) [56]. Similarly, other studies on patients with cancer in the United States and Europe revealed that the seroconversion rates and antibody titers were significantly lower after the first vaccine dose compared to those after the second dose. Much better antibody responses at 3 weeks after the second dose of mRNA SARS-CoV-2 vaccines were observed in Swiss patients [55]. In a larger study, including 200 patients with cancer who had received the full dosing of the mRNA-1273, BNT162b2, or Ad26.COV2.S vaccine, patients with solid tumors had a high seroconversion rate including those who receive ICIs (97%) [54]. Recently, Gounant et al. reported the results for 306 French patients with thoracic cancer from a prospective observational COVID-19 VAC-OH study investigating the SARS-CoV-2 vaccination effectiveness (mainly mRNA-based vaccines) [69]. The results supported the efficacy of mRNA COVID-19 vaccines in patients with thoracic cancer. Thirty patients (1%) with persistent low antibody titers received a third vaccine dose, resulting in an 88% immunization rate.

Data from early studies in Spring 2021 did not include patients potentially infected with the Delta variant, so it was not possible to fully evaluate efficacy after a second dose of the vaccine in Delta-dominated cases [79,80]. The emergence of Omicron as the dominant strain further complicated matters. Valanparambil et al. reported that patients with lung cancer had a 79-fold lower neutralizing response to Omicron compared to individuals without cancer after two doses of an mRNA vaccine [81]. Fendler et al. reported that neutralizing antibodies against Omicron are rarely detected after two vaccine doses, but approximately 50% of patients have detectable neutralizing antibodies after a third dose [82]. Data from recent studies confirmed that the booster vaccination in these patients is well tolerated, and the ability to neutralize variants of concern was reported [83,84]. The use of heterologous booster vaccines in patients with cancer is also complicated, precluding any meaningful conclusions on the most effective vaccine combination.

Patients with cancer often have discordant humoral and cellular responses to vaccination, a situation that is rarely observed in other populations [85]. During 2021, the role of cellular responses to vaccines became clearer. Specifically, T-cell responses to vaccination against COVID-19 are detectable in 46–79% of patients with solid tumors. Receiving treatment for cancer, recent chemotherapy, or steroid use within 15 days of vaccination has been associated with reduced T-cell responses to vaccination, as seen in the assessment of humoral response [56,85,86]. T-cell responses to vaccination are less affected by B-cell-depleting therapies [59]. Specifically, T-cell responses, especially CD8+ responses, have been detected in patients receiving B-cell-depleting therapies who subsequently developed COVID-19, even in the absence of humoral responses, indicating that T-cell responses alone can provide protection against poor outcomes [87,88].

Data from studies involving patients with cancer so far indicate that the adverse events did not differ based on registration with the various vaccine platforms. The most common adverse events reported were soreness or pain at or around the injection site (63% of vaccines), local swelling (9%), muscle pain (34%), fatigue (34%), headache (16%), fever (10%), chills (10%), and gastrointestinal events (10%). Of course, there is still no knowledge of the long-term adverse effects of COVID-19 vaccines in individuals with and without cancer due to the very short observation time [57,60]. Patients receiving immunocheckpoint inhibitors were at possible additional risk of developing irAEs after the COVID-19 vaccination, but this was not confirmed [57]. Radiation phenomena, such as pneumonitis or dermatitis, have also been described following the COVID-19 vaccination and can sometimes significantly complicate discernment; awareness of this complication is important to avoid mistaking such phenomena as adverse effects of cancer therapy [89,90].

## 5. Discussion and Open Questions

The question of the ultimate comorbidity between cancer and COVID-19 remains open and unexplored, mainly due to a lack of reliable databases and comprehensive clinical measurements and/or follow-ups among relevant groups of patients. The problem of comorbidity is indeed complex, especially from the perspective of therapy using immune checkpoint inhibitors combined with the eventual co-application of genetic constructs aimed at coding a viral spike protein, particularly mRNA constructs. Given that the adequate monitoring time frame is a crucial factor in the evaluation of any outcome, it is impossible to draw final conclusions. Consequently, we can only consider rational scenarios. While the idea of vaccination can only be positively evaluated from a medical point of view, the products used for this purpose should be very carefully studied and monitored over the long term. Indeed, lung cancer, due to its immune consequences, causes a depletion of the immune system, which favors the development and progression of COVID-19. From this perspective, it can be assumed that vaccination, assuming it works, will have a positive effect because a vaccine stimulates a part of the body’s immune functions. In this context, it would be particularly relevant to monitor, over a longer term, the effects of genetic constructs used for COVID-19 vaccination on the immune system components, as recent papers also showed negative impacts on immune functions, especially in the field of natural immunity constituents. Therefore, it is necessary to consider both scenarios and, over time, observe the outcomes of comorbidities, which are currently lacking objective data due to the short timeframe of the observations.

Of particular concern is the effect of anticancer treatment with immune checkpoint inhibitors on the immune response among SARS-CoV-2 cancer patients. If we introduce a new variable into this consideration through vaccination with mRNA or a vector vaccine, we obtain an equation with multiple unknowns that are not easy to solve. The hypothesis outlined in this paper is that patients with lung cancer have or may have an increased risk of developing a more severe form of COVID-19. This assumption is based on the fact that the immune situation of lung cancer patients is compromised and that SARS-CoV-2 infection will have negative consequences on the development of the disease. The cross-section of this issue, through different types of cancer, shows that these results are not conclusive, likely due to the short observation timeframe. Another unknown factor is the presence of other comorbidities in patients such as diabetes, cardiovascular disease, obesity, autoimmune diseases, and other immune deficiencies.

Due to the topical effects of SARS-CoV-2, lung cancer patients are expected to be more vulnerable to COVID-19, as corroborated by several studies previously mentioned in this paper. The reported official data on the mortality rate proved to be significantly higher than the realistic estimations. Unfortunately, we will not be able to obtain proper conclusions solely from this information. For example, the increased general mortality data for patients with lung cancer and COVID-19 may also be due to inadequate COVID-19 treatment, especially in the early stages of the disease [91]. In addition, no comprehensive data are available on the number of patients who died suffering from both lung cancer and COVID-19, and were vaccinated with currently used genetic constructs. Therefore, we cannot consider the available data to be completely appropriate for the final conclusions on this matter.

There are also other open questions regarding lung cancer and COVID-19. One of these questions concerns whether SARS-CoV-2 infects lung cancer cells and whether the infection is important in the pathogenesis of the COVID-19 disease. This question can be answered directly via the extermination of human lung cancer cells in a virus culture or infected tissue. The path of the virus itself (or viroid) can also be synthesized under laboratory conditions. The theoretical infection of tumor cells with SARS-CoV-2 could determine whether the virus can have dual effects on cancer cells. The protein spike is expected to have a negative effect on the outcome of the disease and on the tumor itself. Herein, we should bear in mind that one of the strategies to combat cancer is the introduction of a virus [92] or bacteria [93] into the cell and, consequently, the cytopathic effect on the tumor.

Another question is whether a SARS-CoV-2 infection of the lungs, in otherwise healthy individuals, affects the future development of lung cancer. The hypothetical answer to this question is very likely negative. Namely, to date, there is no data on whether SARS-CoV-2 is genotoxic or carcinogenic, so more research is necessary in this area. This need is even more significant for the current gene constructs used as vaccines. Some data from the literature raised the question of an increased incidence of certain cancers among vaccinated individuals [94], but for earlier claims, more time is required to obtain conclusive data.

Another important question is whether COVID-19 pulmonary disease affects the clinical behavior of lung cancer in individual patients. That question is perhaps the easiest to answer among the open issues on this subject. At the same time, the situation should be viewed objectively, and patients who received the vaccine should be excluded from the study. Again, there remains a hypothetical question on whether SARS-CoV-2 infects cancer cells and, if yes, at what time. From the point of view of the immune function, the processing of the immune response to infection and against the autoimmune response could be triggered. In principle, a concomitant immune response is expected.

The next important question is whether clinical characteristics and therapy of lung cancer affect the development and severity of COVID-19 and anti-SARS-CoV-2 immune responses. For this question, it is necessary to respond solely by considering each patient individually. Certainly, one of the main problems for anti-COVID-19 vaccination is the absence of personalized criteria and the consideration of each patient individually. Finally, we do not know whether we can improve anti-SARS-CoV-2 immune responses in patients with lung cancer. If the hypothetic superiority of natural immunity over that induced by vaccines proves to be correct, then this question loses its meaning. In any case, the answer to these questions could be given partially through a comparison of cancer and influenza cases treated by similar vaccine products. Until we consider the paradigm of personalized vaccination (not only general vaccination but also cancer vaccination) we will face this problem [95,96].

In light of new knowledge about the effects of the genetic products used for COVID-19 vaccination, the claim that these products are effective and safe in the long term is very vague and scientifically unsupported. Such claims require a longer time frame, comprehensive and independent clinical studies, reliable databases, and input data. The available data reported to date mostly originated from heterogeneous patient cohorts, and published information does not contain all observed clinical parameters, which makes it challenging to draw robust conclusions on the optimal approach for COVID-19 vaccination in patients with cancer.

The composition of mRNA vaccines may also be important for lung cancer patients. These products, indeed, contain modified mRNA coding the spike protein. The idea of these products is to hide mRNA from cellular defenses, extend the half-life of spike proteins, and cause greater production of the spike protein within the cells. Experimental and clinical data, however, show a significant difference in the immune response to the mRNA vaccine relative to the natural immune response to SARS-CoV-2. Namely, vaccination with these products seems to damage the signaling of interferon I, which has negative consequences for human health, notably the occurrence of various forms of cancer in patients that received these products [94]. The authors of a previous study pointed to the appearance of disorders in the regulatory control of protein synthesis and the control of cancer. Earlier this year, it was found that the spike protein created by the COVID-19 vaccines remains in the body much longer than initially assumed and at levels higher than those of severely ill COVID-19 patients [97].

The pandemic’s urgency placed the health-care system under high pressure, and the hope for vaccines against COVID-19 was high, especially for certain high-risk groups of patients, such as those with lung cancer. After more than 1 year of genetic products being available for COVID-19 vaccination, especially for at-risk patients, a need has been observed for a more detailed evaluation of data on COVID-19 and lung cancer comorbidity among vaccinated and unvaccinated cohorts of patients. Relevant therapeutic measures and procedures should also be included in this evaluation. The need for more reliable databases including data on widely measured immune parameters in COVID-19 lung cancer patients, including in vaccinated COVID-19 lung cancer patients, as well as spike protein level measurements in the blood, may shed some light on the previously discussed open questions in this field. Thus, individual monitoring and therapeutic adjustments can be advised as a safe approach for the management of COVID-19 lung cancer patients.

## Figures and Tables

**Table 2:** Recent studies of COVID-19 vaccine efficacy in patients with cancer.

Population	Publication Date	Cancer Type	Vaccine	Seroconversion Rate after 2nd Dose	Comment	Reference
Israel	May 2021	Solid	Pfizer BNT162b2	90%		[53]
USA	May 2021	Solid	Pfizer BNT162b2	80%		[61]
UK	June 2021	Various	Pfizer BNT162b2	79%		[6]
Israel	July 2021	Various	Pfizer BNT162b2	86%		[52]
USA	July 2021	Hem (MM)	Pfizer BNT162b2 Moderna mRNA-1273	77%		[62]
Israel	July 2021	Hem (CLL)	Pfizer BNT162b2	43%		[63]
USA	August 2021	Various	Pfizer BNT162b2, Moderna mRNA-1273, Johnson & Johnson Ad26.COV2.S	95% 94% 85%	Solid cancer 98%, ICIs 97%, Hem 85%, antiCD20+ 70%, stem cell transplants 73%	[54]
France	August 2021	Solid and hem	Pfizer BNT162b2 Moderna mRNA-1273 Janssen	94% solid 85% hem		[64]
Switzerland	August 2021	Various	Pfizer BNT162b2, Moderna mRNA-1273	93–95%		[55]
USA	August 2021	Hem	Pfizer BNT162b2 Moderna mRNA-1273	75%		[65]
France	September 2021	Solid	Pfizer BNT162b2	95%		[51]
Netherlands	September 2021	Solid	Moderna mRNA-1273	97–100%	Chemotherapy 97.4% IO 99.3% Chemoth + IO 100%	[66]
Denmark	September 2021	Solid and hem	Pfizer BNT162b2 Moderna mRNA-1273	93% solid 66% hem		[67]
UK	September 2021	Solid and hem	Pfizer BNT162b2 AstraZeneca	85% solid 54% hem		[56]
USA	November 2021	Solid and hem	Pfizer BNT162b2 Moderna mRNA-1273	72–84% solid 62–85% hem		[68]
France	February 2022	Thoracic	Pfizer BNT162b2	94%		[69]

mRNA-1273 (Moderna, Cambridge, MA, USA), BNT162b2 (Pfizer BioNTech, New York, NY, USA), and Ad26.COV2.S (Johnson & Johnson Janssen, Leiden, The Netherlands), hem—hematologic; CLL—chronic lymphocytic leukemia, MM—multiple myeloma.

## Data Availability

Not applicable.
